# Virtual reality and psychedelics: new perspectives and new possibilities in the treatment of alcohol use disorder

**DOI:** 10.3389/fpsyt.2025.1448043

**Published:** 2025-11-13

**Authors:** Scott Matthews, Antonino Greco, Clara Rastelli, Anahita Bassir Nia

**Affiliations:** 1 Department of Psychiatry, Veterans Administration Medical Center, West Haven, CT, United States; 2 Department of Psychiatry, Yale University School of Medicine, New Haven, CT, United States; 3 Department of Neural Dynamics and Magnetoencephalography, Hertie Institute for Clinical Brain Research, Center for Integrative Brain Research, University of Tubingen, Tubingen, Germany; 4 MEG Center, University of Tubingen, Tubingen, Germany; 5 Werner Reichardt Centre for Integrative Neuroscience, University of Tubingen, Tubingen, Germany; 6 Magnetoencephalography Centre, University of Tubingen, Tubingen, Germany; 7 Hertie Institute for Clinical Brain Research, Centre for Integrative Neuroscience, University of Tubingen, Tubingen, Germany; 8 Department of Psychology and Cognitive Science, University of Trento, Rovereto, Italy; 9 Clinical Neuroscience Research Unit, Connecticut Mental Health Center, New Haven, CT, United States

**Keywords:** virtual reality, psychedelic assisted therapy (PAT), alcohol use disorder (AUD), psychedelic placebo, psychedelic phenomenology, *DeepDream*

## Abstract

Psychedelic-assisted therapy is a remarkably promising treatment for substance use disorders and for alcohol use disorder (AUD) in particular. Research supporting psychedelics as a safe and effective pharmacotherapy for AUD and its comorbid psychiatric conditions dates from the late1940s and includes over 2,000 published studies. There are, however, challenges to the clinical implementation of psychedelics on a scale appropriate to a highly prevalent disease. Virtual reality applications can aid in meeting these challenges. Studies have shown that virtual reality simulations of psychedelic phenomenology (VRP) can replicate neurophysiological and behavioral markers of classic serotonergic psychedelics when viewed by healthy subjects. The results of these studies suggest VRP may have utility as an adjunct to or replacement for aspects of psychedelic-assisted therapy. Here, we introduce four potential clinical applications for VRP in psychedelic-assisted therapy: to prepare for psychedelics, to extend and enhance their efficacy, to facilitate integration following psychedelic dosing, and to standardize the set and setting. VRP may also have application to clinical research in psychedelics as a placebo and as a tool for interrogating the subjective experience of psychedelics in the therapeutic setting. Integrating virtual reality into psychedelic therapy and research holds the promise of new possibilities for psychedelic treatment and new pathways to their implementation.

## Background

Excessive alcohol consumption is a public health crisis in the United States. As of 2022, 29.5 million people in the United States (10.5% of the population over 12 years old) were estimated to have alcohol use disorder (1). Alarmingly, the situation appears to be getting worse. An analysis of death certificates from 2019 and 2020 showed that deaths involving alcohol rose from approximately 79,000 to more than 99,000, a 25.5% increase (2). In a longer view, a 2023 study showed alcohol-induced mortality in the US increased by 50% from 1999 to 2022 (3). Compounding the problem, only 7.6% of Americans with past-year alcohol use disorder (AUD) received treatment (4). While medication-assisted treatment is considered state of the art, only three medications (Naltrexone, Acamprosate, and Disulfiram) have been approved by the FDA, and an estimated 70% of individuals engaged in treatment relapse within 12 months (1). There is an urgent need for more effective and accessible treatment, including an increased number of pharmacological options. This need has motivated a search for novel or underutilized therapies.

## A brief history of psychedelics and AUD

The peculiar history of psychedelics qualifies them as both novel and underutilized. Early research supports their efficacy in the treatment of AUD and its comorbid psychiatric disorders. Between 1948 and 1976, the treatment of an estimated 40,000 subjects was documented in over 1,000 published studies (5, 6). The subjects in these studies were diagnosed with addiction and psychiatric disorders and treated with classic serotonergic hallucinogens, primarily lysergic acid diethylamide (LSD). In a 2012 meta-analysis, Krebs and Johansen looked at six randomized controlled trials with 636 subjects conducted during this period. Subjects in these trials were treated for AUD with LSD. The authors found a significant reduction in alcohol misuse versus placebo, with a 1.96 odds ratio (OR), superior to that of naltrexone (0.69) and all other currently available pharmacotherapies for AUD (7).

So why weren’t psychedelics incorporated into the armamentarium of addiction medicine? The reasons for this had everything to do with politics and nothing to do with science. When President Richard Nixon prevailed upon Congress to pass the Controlled Substances Act of 1970, it became illegal to possess classic serotonergic hallucinogens like LSD and psilocybin, and they were classified as Schedule I drugs. This effectively ended academic psychedelic research in the United States (6, 8). It would be nearly a quarter-century before studies of intravenous DMT in healthy volunteers by Strassman et al. in the mid-1990s paved the way for a “second golden age” of psychedelic research beginning in the early 2000s (6, 9–11). Since 2004, over 2000 articles have been published in peer-reviewed journals investigating psychedelics as a treatment for substance use disorders and other psychiatric disorders such as depression (5).

However, nearly all these studies had small sample sizes or did not include a placebo arm. The recently published randomized controlled trial of psilocybin as a treatment for AUD by Bogenshutz, Ross et al. (12) is notable both for its results and its protocol. All participants had a diagnosis of alcohol dependence and engaged in twelve psychotherapy sessions with providers who included a psychiatrist and trained psychotherapists. Participants were dosed with psilocybin during weeks four and eight of the protocol. A significant reduction in the number of heavy drinking days was reported in the psilocybin group between weeks 5 and 36 of the study. These results were consistent with an earlier study (2015) by Bogenshutz et al. and with other studies of psychedelics as a treatment for AUD (8, 13). Like the previous study, this one identified the quality of participant experience, as indicated by scores on the Mystical Experience Questionnaire, as a predictor of clinical efficacy.

## Challenges to clinical translation: placebos, implementation, and study design

### The placebo problem

Bogenschutz, Ross, et al. demonstrated the difficulty of maintaining blinding in psychedelic research. Participants in this randomized, double-blind clinical trial correctly guessed their treatment assignment at the rate of 93.6% for the first session and 94.7% for the second session. As Van Elk and Fried (2023) observed, most PAT trials are not effectively blinded, and “breaking blind is the rule rather than the exception in psychedelic trials.” They noted the absence of tenable placebos and highlighted a finding from a recent systematic review noting that while most psychedelic trials are presented as blinded, blinding was evaluated in only 8 of 81 peer-reviewed studies (14).

### Feasibility of implementation

The results of Bogenshutz, Ross, et al. argue for the efficacy of psychedelics as a treatment for AUD. The study also presents some challenges to implementing PAT on a scale appropriate to a highly prevalent illness. Foremost among these is the resource-intensive nature of the treatment. Participants received 12 weeks of sequenced manualized therapy in addition to two eight-hour monitored dosing sessions. It is difficult to imagine this protocol adopted as an outpatient treatment regimen due to its expense and the critical role played by providers with specialized training (15).

### Study design limitations

The resource-intensive nature of the study protocol is related to a second challenge: establishing a fund of knowledge sufficient to inform evidence-based clinical practice. To date, the study by Bogenshutz et al. has been the only recent larger-scale, randomized, controlled trial of PAT for AUD. While there are a considerable number of small-group peer-reviewed studies, they usually have protocols that vary widely and present challenges to evaluation for clinical translation, including the difficulty of maintaining blinding noted above.

It should also be noted that exclusion and inclusion criteria for studies evaluating PAT for AUD would benefit from standardization. An example of this might use of an alcohol breath test to determine exposure prior to beginning a trial of PAT.

## A role for virtual reality in psychedelic research and treatment

Here, we propose that virtual reality (VR) applications may contribute to meeting the challenges described above. Below, we introduce some of the modalities through which these contributions might be made.

### VR as a treatment modality

VR simulations of psychedelic phenomenology (VRP) modeled on functional aspects of the mammalian visual cortex are an especially promising area of research. *DeepDream* (DD), an algorithm that enables biologically plausible simulation of psychedelic phenomenology, was developed in 2015 by Google engineer Alexander Mordvintsev (16, 17). DD uses pre-trained deep convolutional neural networks (DCNN) to alter images. DCNN image processing parallels that of the mammalian visual cortex, with each layer detecting specific features in an image (18, 19) As information flows through the network, deeper layers combine these features to identify objects and patterns. This identification is possible because DCNN are trained using large-scale image datasets to perform object recognition (20).

Although the DCNN training objective is to forward pass the input image to predict the correct identification, DD instead uses the DCNN to invert the biological image processing model by altering the input image itself. DD provides users the option of clamping processing activity at a user-defined layer in the DCNN and then inverts the information flow so that an input image is changed until the network settles into a stable state (Mordvintsev et al., 2015; Nguyen et al., 2016). This involves changing the image rather than changing the network to match the features of the image with what is represented in the target layer. This results in an image shaped by what the network “expects” to see at the level of detail determined by the clamped layer. Clamping at lower levels will produce images altered in geometric patterns while clamping at higher levels imposes object-like features on the input resembling complex hallucinations (21).

A 2017 paper by Suzuki et al. described using DD to create what the authors called a “Hallucination Machine” to produce biologically realistic visual hallucinations. The Hallucination Machine applied the DD algorithm to each frame of a prerecorded panoramic video presented using a head-mounted device (Oculus Rift 2). Subjects viewed either a control video of nature scenes or the DD panoramic video. After viewing the videos, participants completed a modified version of the Altered States of Consciousness questionnaire (ASC) (22). For participants exposed to DD video, responses on the ASC were similar to subjects in another study who had taken psilocybin for the items assessing the intensity, patterns, feeling of profound inner peace, strangeness, vividness, and spiritual or mystical quality experienced while viewing (23).

This was followed by a 2021 study by Greco et al., providing neurophysiological evidence of the similarity of DD exposure and psychedelic experience following dosing with a hallucinogen. The authors took EEG readings of participants while viewing unaltered video footage and the same footage altered by DD on a computer screen. DD viewing produced increased functional connectivity in the gamma band and increased global entropy. These findings were comparable to those of Roseman, Leech et al. and Carhart-Harris, Leech et al. for subjects under the influence of psilocybin (24–26).

In 2022, a paper by Rastelli et al. provided further evidence of the capacity of DD to simulate psychedelic phenomenology and reproduce its effects (27). One of the central therapeutic markers of psychedelic-assisted therapy is an increase in cognitive flexibility (CF) (28, 29). CF is a central aspect of cognition, permitting adaptation to changing environmental demands, and is markedly decreased in both depression and addiction (30, 31). In Rastelli et al., the authors measured participants’ cognitive flexibility through task performance after exposure to virtual reality panoramic videos generated by the DD algorithm. They found that exposure to simulated visual hallucinations increased cognitive flexibility compared to naturalistic (control) videos. This gain in cognitive flexibility was similar to that measured following dosing with psilocybin (32) and was recently replicated in another study using immersive simulated hallucinations (40). Furthermore, another recent study found that perturbing perceptual phenomenology with simulated visual hallucinations modulated high level cognition in human subjects (41, 42).

Taken together, these two studies suggest that virtual reality simulations of psychedelics (VRP) can replicate some of the neurophysiological outcomes of psychedelics in healthy subjects. This argues that VR-simulated hallucinations may be worth investigating as treatments for the same conditions – including alcohol use disorder, nicotine use disorder, and depression – for which psychedelic-assisted therapy has shown efficacy. To date, no study has tested the efficacy of VRP using DD as a treatment for these and other conditions, and no study has trialed VRP against a classic serotonergic psychedelic for any indicated condition.

### VR as preparation for psychedelic therapy

Apart from promise as a stand-alone treatment, virtual reality applications may have utility as part of a psychedelic-assisted therapy protocol. With ketamine infusions, providers report that patients naïve to the drug are anxious about starting treatment, regarding the experience as novel and potentially discomforting (33). It is reasonable to expect that this anxiety may extend to psychedelic therapies as a whole for some individuals. Providing such patients with a virtual reality simulation of the therapy prior to dosing may reduce anxiety and allow the patient to benefit from the treatment. In the event the simulation provokes anxiety or another deleterious response, VRP viewing can be stopped at will, unlike the effects of a psychedelic.

Exposure to nature-based videos that do not attempt to simulate psychedelic experience has been investigated as an option for enhancing set and setting for participants in PAT with AUD. Heinzerling et al. (2023) used “Visual Healing”, a nature-themed video, and explained their choice this way: “We chose a nature theme for the video as use of psychedelics in traditional healing rituals and outside of clinical trials often occurred in natural and outdoor settings and a greater feeling of connectedness to oneself, others, and the natural world is consistently reported following psychedelic experiences.” Study participants watched a three minute introductory video during a preparation session and forty-two minute and fifteen minute videos at the opening and closing of the psilocybin dosing session. Primary outcomes in this randomized controlled trial were increases in heart rate and blood pressure pre- and post-psilocybin dosing. Exploratory outcomes included alcohol use, psychedelic effects, stress, and anxiety. The investigators found that participants viewing nature themed video, Visual Healing, had a peak increase in post-psilocybin blood pressure significantly lower than participants viewing a control video. There were no statistically significant differences in exploratory outcomes between the control and experimental groups.

While this review focuses on virtual reality simulations of psychedelics experience for a targeted therapeutic intervention (AUD), there have been more general and theoretical explorations of psychedelic simulations. Some of these fall under the heading of “Cyberdelics”, conceived as a union of digital technology and psychedelics. Cyberdelics are designed to produce altered states of consciousness distinguished by the experience of awe and transcendence associated with classic serotonergic psychedelics. Cyberdelic pioneers characterized immersive virtual reality applications as the nearest analogue to dosing with psychedelics and promoted cyberdelics as a utopian project with the potential to produce “such beauty, fascination and depth that mankind will be seduced away from mass suicide” (Lanier 2017). To date, there have been no documented attempts to harness applications identified as cyberdelic for social change or therapeutic applications.

While cyberdelics have not been evaluated as a means of promoting pro-social change at population scale, researchers have attempted to replicate aspects of psychedelic experience using VR in group therapy. Glowacki et al. (2022) implemented a VR application called “Is-Ness distributed”in a group setting. The study used healthy volunteers at multiple sites simultaneously using the application. The application depicted the bodies of participants merging in a shared “virtual space”, with the primary outcome of the study being induction of a “self-transcendent experience”. The authors used the Mystical Experience Questionnaire (MEQ 30), the Ego Dissolution Index (EDI), the Communitas Questionnaire, written reflection, and semi-structured group discussion to assess such experiences. They reported responses for self-transcendent experiences comparable to low doses of psilocybin (18–20 mg) on these assessments.

Although VRP applications are the only ones rigorously evaluated for simulation of psychedelic phenomenology, there are other commercially available virtual reality applications attempting to replicate beneficial or pleasant aspects of the subjective experience of psychedelics. Some of these were designed as psychotherapeutic interventions. They include Liminal VR, Cosmic Flow, and Deep States. These applications are primarily focused on relieving stress, promoting mindfulness, and inducing states of relaxation (Casu et al, 2024).

### Using VR to extend and enhance the effects of psychedelics

VRP may also be able to extend the duration of effect for psychedelic-assisted treatment. There is evidence that the efficacy of psychedelic treatments attenuates over time, with estimates ranging from 6 weeks to 9 months for classic serotonergic psychedelics (34). If brief VRP sessions, administered either in a clinic or at home by patients, could extend the therapeutic effect’s duration, this would make the treatment more economical and, hence, more accessible.

VRP may also make it possible to target areas of therapeutic importance in the psychedelic experience. Subjects commonly report between one and three significant events in a psychedelic dosing session. These may include episodes ranging from an encounter with a deceased relative to a visit to another planet, but subjects regard each as having thematic content and providing therapeutic benefit (35). Frequently, however, subjects report difficulty recalling these events in sufficient detail to discuss them in psychotherapeutic sessions following dosing (15, 36). VRP may make it possible to stimulate recall of these events and make the material accessible in psychotherapy.

If VRP is shown to provide therapeutic benefit, it may also be useful as an intervention for individuals with AUD who have benefitted from PAT but experienced relapse. For patients with a history of relapse following PAT, VRP sessions could be scheduled at intervals preceding periods of increased relapse risk.

### VR as a tool for interrogating the role of subjective experience in PAT

There is a debate among researchers as to whether the therapeutic effect of psychedelics is mediated through the unique subjective experience made possible by these substances. Yaden et al., Bogenshutz, Ross, et al., and others argue that the subjective experience is necessary for the therapeutic effect of psychedelics and that favorable outcomes may be attributed to aspects of the subjective experience, particularly those indicating profound spiritual experiences (12, 35). Olson et al. and others contend that the correlation between subjective psychedelic experience and therapeutic outcome does not equal causation, arguing that therapeutic efficacy depends only on the neuroplasticity induced by psychedelic agents binding to receptors (37). While each side in the debate can cite evidence supporting their contentions, no study to date has evaluated the efficacy of subjective experience alone in the treatment of AUD and depression. A trial of VRP for these conditions would make such a determination possible. Alternatively, if the experiment demonstrated therapeutic efficacy for VRP, then full or partial substitution of VRP for psychedelic agents could be considered.

### Using VR to standardize set and setting

As noted above, nearly all the evidence supporting the use of psychedelic-assisted therapy has come from small studies, often open-label, and with a variety of protocols, making comparison difficult. Absent a massive infusion of research funding, it is unlikely that these limitations can be overcome in the near future.

However, VR may be able to mitigate some of these limitations. One approach to compensating for the absence of large randomized controlled trials is establishing a standard protocol for small (10–30 subjects) registered studies. To date, one of the principal sources of variation in study protocols has been their approach to dosing “set” and “setting.” Here, “set” refers to the global mental state of a patient at the time of dosing and encompasses mood, expectations, beliefs, anxieties, and therapeutic intentions. “Setting” refers to the physical and social environment of the dosing session. It includes the session location, sensory elements (lighting, temperature, ambient noise), and the presence of patient care team members, usually physicians and psychotherapists. The set and setting of dosing sessions influences the subjective experience and therapeutic efficacy of psychedelic therapies (15, 35). In studies published to date, participants are usually prepared for the dosing sessions with one or more psychotherapy sessions (38, 39). There is no standardized training for therapists working with psychedelics and no validated assessment of a patient’s “set” and readiness for dosing. Neither are there guidelines for settings, and these can vary from intensively controlled environments with therapists present to unmodified treatment rooms with minimal therapeutic resources.

Here, VR can play an essential role in both the set and the setting. VRP can be used to preview the subjective experience of psychedelic dosing for subjects, standardizing the set for dosing. Following this, VR applications with head-mounted displays (HMD) and noise-blocking headphones can also be used to standardize settings, obviating the need for a customized dosing area.

### VR as a placebo in psychedelic research

Blinding is another area of variation in PAT studies and a frequent target of criticism. Many studies of psychedelics are open-label or lack a tenable blinding procedure (14, 35). Here, a credible VRP, albeit without a therapeutic effect, may be a way of effectively blinding study participants.

### VR modalities to enhance integration in PAT

While these uses of VR, whether as a substitute for, a complement to, or an integral part of PAT, are worth exploring, the most intriguing possibility may be the potential for VR to facilitate interrogation of the mechanisms through which PAT exerts therapeutic effects. As noted above, VR, in contrast to psychedelic agents, has a period of activity that can be stopped and started according to the goals of a study. It can simulate fundamental aspects of psychedelic phenomenology and produce representations of almost any conceivable image, pattern, or geometric configuration with recent developments in artificial intelligence. These capabilities enable VR to replicate the experience of psychedelic intoxication in a way that allows for interrogation in real-time. This interrogation could directly correlate highly personal, often ineffable experiences with electrophysiological states and therapeutic outcomes. Achieving this correlation could facilitate post-dosing therapeutic integration of experiences PAT patients have characterized as profoundly meaningful and often with spiritual import. It may also allow for the continuation of the subjective experiences patients value without impacting implementation feasibility.

### VR and risk

While VR simulations involve none of risks commonly associated with psychedelic dosing, it is not without risk as a therapeutic modality. The use of any virtual reality device comes with the possibility of virtual reality sickness, a variant of motion sickness. Virtual reality sickness commonly presents with eyestrain, nausea, headache, pallor, sweating, and fatigue. After the sufferer removes the VR viewing device, virtual reality sickness generally resolves within thirty minutes. There is no indication that individuals with AUD would be more susceptible to virtual reality sickness than individuals without the disorder.

The risks posed by persons attempting to use VRP to treat AUD without medical supervision are more difficult to assess. It may be the best preventive measure for this would be to license the software solely to health care providers.

## Conclusion

Psychedelics are unique among pharmacotherapies for AUD and its frequent comorbid condition, depression, and promise dramatic improvement after only one or two doses, with efficacy continuing for up to six months and possibly even longer. They also have the potential for transdiagnostic efficacy, raising the possibility that a single treatment session might provide relief for anxiety, cluster headaches, and nicotine addiction. Patients frequently characterize psychedelics as having benefits additional to treatment for an identified condition, attributing profound spiritual insight and increased empathy to dosing (38).

The unique properties of psychedelics also complicate their path to clinical implementation. Current approaches to clinical translation, medication approval, and incorporation into evidence-based practice were developed for treatments whose attributes have little in common with psychedelics. These approaches rely on systems optimized to evaluate medications with defined mechanisms of action targeted to specific diseases whose efficacy can be measured through a limited number of clinical outcomes. The assumption is that the medication in question will work roughly the same in every patient indicated for treatment and that adverse effects and clinical outcomes are consistent and dose-dependent.

Proponents of psychedelics argue that the features making them the square peg in the round hole of medication protocols are also responsible for their distinguishing therapeutic efficacy. They contend that providing patients with similar conditions with different experiences and a range of therapeutic outcomes is an advantage, evidence of the multifaceted functionality of psychedelics and their potential role in personalized treatment regimens (35). While this argument may have merit, it is unlikely that medication evaluation and implementation systems would be reconfigured to accommodate a single class of agents solely based on clinical efficacy.

Still, none of these considerations diminish the urgency of implementing new treatments for AUD or the therapeutic promise of PAT. It is worth noting in this context that the term “psychedelic” is compounded of Greek words for “mind” (psyche) and “to manifest” (deloun). The signal contribution of VR to the development of psychedelic therapy may be providing new perspectives on how the mind manifests under psychedelics and the ramifications of this manifestation. In this capacity, it can examine an issue fundamental to PAT: Do psychedelics induce a unique mental state, one possible only through a class of psychoactive substances? Or do they facilitate an intensification of consciousness, which might be accessible through intentional practice without using any drug? If establishing a reciprocal heurism between VR and psychedelics can constructively address this question, it may lead to new possibilities for PAT and new pathways to clinical implementation.

## References

[1] NIAAA NIoAAaA . Alcohol facts and statistics (2024). Available online at: https://www.niaaa.nih.gov/alcohols-effects-health/alcohol-topics/alcohol-facts-and-statistics (Accessed February 11, 2024).

[2] Esser MB SA Liu Y Naimi TS . MMWR Alcohol use deaths.pdf. (2024). (Bethesda, Maryland, USA: National Institute on Alcohol Abuse and Alcoholism).

[3] Maleki N Yunusa I Karaye IM . Alcohol-induced mortality in the USA: trends from 1999 to 2020. Int J Ment Health Addict. (2023) 6:1–13. doi: 10.1007/s11469-023-01083-1, PMID: 37363762 PMC10243241

[4] Alcoholism NIoAAa . Prevalence of past-year alcohol use treatment. In: Alcohol treatment in the United States (2024) (Bethesda, Maryland, USA: National Institute on Alcohol Abuse and Alcoholism). Available online at: https://www.niaaa.nih.gov/alcohols-effects-health/alcohol-topics/alcohol-facts-and-statistics/alcohol-treatment-united-states.

[5] Mitchell JM Anderson BT . Psychedelic therapies reconsidered: compounds, clinical indications, and cautious optimism. Neuropsychopharmacology. (2024) 49:96–103. doi: 10.1038/s41386-023-01656-7, PMID: 37479859 PMC10700471

[6] Nichols DE Walter H . The history of psychedelics in psychiatry. Pharmacopsychiatry. (2021) 54:151–66. doi: 10.1055/a-1310-3990, PMID: 33285579

[7] Krebs TS Johansen P . Lysergic acid diethylamide (LSD) for alcoholism: meta-analysis of randomized controlled trials. J Psychopharmacol. (2012) 26:994–1002. doi: 10.1177/0269881112439253, PMID: 22406913

[8] Doblin RE Christiansen M Jerome L Burge B . The past and future of psychedelic science: an introduction to this issue. J Psychoactive Drugs. (2019) 51:93–7. doi: 10.1080/02791072.2019.1606472, PMID: 31132970

[9] Strassman RJ Qualls CR . Dose-response study of N,N-dimethyltryptamine in humans. I. Neuroendocrine, autonomic, and cardiovascular effects. Arch Gen Psychiatry. (1994) 51:85–97. doi: 10.1001/archpsyc.1994.03950020009001, PMID: 8297216

[10] Strassman RJ Qualls CR Uhlenhuth EH Kellner R . Dose-response study of N,N-dimethyltryptamine in humans. II. Subjective effects and preliminary results of a new rating scale. Arch Gen Psychiatry. (1994) 51:98–108. doi: 10.1001/archpsyc.1994.03950020022002, PMID: 8297217

[11] Strassman RJ . Human psychopharmacology of N,N-dimethyltryptamine. Behav Brain Res. (1996) 73:121–4. doi: 10.1016/0166-4328(96)00081-2, PMID: 8788488

[12] Bogenschutz MP Ross S Bhatt S Baron T Forcehimes MAA Laska E . Percentage of heavy drinking days following psilocybin-assisted psychotherapy vs placebo in the treatment of adult patients with alcohol use disorder: A randomized clinical trial. JAMA Psychiatry. (2022) 79:953–62. doi: 10.1001/jamapsychiatry.2022.2096, PMID: 36001306 PMC9403854

[13] Bogenschutz MP Forcehimes AA Pommy JA Wilcox CE Barbosa PC Strassman RJ . Psilocybin-assisted treatment for alcohol dependence: a proof-of-concept study. J Psychopharmacol. (2015) 29:289–99. doi: 10.1177/0269881114565144, PMID: 25586396

[14] van Elk M Fried EI . History repeating: guidelines to address common problems in psychedelic science. Ther Adv Psychopharmacol. (2023) 13:20451253231198466. doi: 10.1177/20451253231198466, PMID: 37766730 PMC10521293

[15] Bogenschutz MP Forcehimes AA . Development of a psychotherapeutic model for psilocybin-assisted treatment of alcoholism. J Humanistic Psychol. (2016) 57:389–414. doi: 10.1177/0022167816673493

[16] Mordvintsev A Olah C Tyka M . Inceptionism: going deeper into neural networks. (2015). Available online at: https://research.google/blog/inceptionism-going-deeper-into-neural-networks/?_gl=1*1xhp6ry*_ga*MTU4MTg2NDkzNC4xNzUzNzkwMTQ2*_ga_163LFDWS1G*czE3NTM3OTAxNDUkbzEkZzAkdDE3NTM3OTAxNDgkajU3JGwwJGgw. (Accessed November 4, 2023).

[17] Nguyen A Dosovitskiy A Yosinski J Brox T Clune J . Synthesizing the preferred inputs for neurons in neural networks via deep generator networks. Adv Neural Inf Process Systems. (2016) 29. doi: 10.48550/arXiv.1605.0930

[18] LeCun Y Bengio Y Hinton G . Deep learning. Nature. (2015) 521:436–44. doi: 10.1038/nature14539, PMID: 26017442

[19] Yamins DLK Hong H Cadieu CF Solomon EA Seibert D DiCarlo JJ . Performance-optimized hierarchical models predict neural responses in higher visual cortex. Proc Natl Acad Sci. (2014) 111:8619–24. doi: 10.1073/pnas.1403112111, PMID: 24812127 PMC4060707

[20] Krizhevsky A Sutskever I Hinton GE . ImageNet classification with deep convolutional neural networks. Adv Neural Inf Process Systems. (2012) 25(6):84–90. doi: 10.1145/3065386

[21] Al-Khazraji L . A systematic review of deep dream. Iraqi J Computer Communication Control System Eng. (2023) 5(2):192–209.

[22] Suzuki K Roseboom W Schwartzman DJ Seth AK . A deep-dream virtual reality platform for studying altered perceptual phenomenology. Sci Rep. (2017) 7:15982. doi: 10.1038/s41598-017-16316-2, PMID: 29167538 PMC5700081

[23] Muthukumaraswamy SD Carhart-Harris RL Moran RJ Brookes MJ Williams TM Errtizoe D . Broadband cortical desynchronization underlies the human psychedelic state. J Neurosci. (2013) 33:15171–83. doi: 10.1523/JNEUROSCI.2063-13.2013, PMID: 24048847 PMC6618409

[24] Greco A Gallitto G D’Alessandro M Rastelli C . Increased entropic brain dynamics during deepDream-induced altered perceptual phenomenology. Entropy (Basel). (2021) 23:839. doi: 10.3390/e23070839, PMID: 34208923 PMC8306862

[25] Roseman L Leech R Feilding A Nutt DJ Carhart-Harris RL . The effects of psilocybin and MDMA on between-network resting state functional connectivity in healthy volunteers. Front Hum Neurosci. (2014) 8:204. doi: 10.3389/fnhum.2014.00204, PMID: 24904346 PMC4034428

[26] Carhart-Harris RL Leech R Hellyer PJ Shanahan M Feilding A Tagliazucchi E . The entropic brain: a theory of conscious states informed by neuroimaging research with psychedelic drugs. Front Hum Neurosci. (2014) 8:20. doi: 10.3389/fnhum.2014.00020, PMID: 24550805 PMC3909994

[27] Rastelli C Greco A Kenett YN Finocchiaro C De Pisapia N . Simulated visual hallucinations in virtual reality enhance cognitive flexibility. Sci Rep. (2022) 12:4027. doi: 10.1038/s41598-022-08047-w, PMID: 35256740 PMC8901713

[28] Murphy-Beiner A Soar K . Ayahuasca’s ‘afterglow’: improved mindfulness and cognitive flexibility in ayahuasca drinkers. Psychopharmacol (Berl). (2020) 237:1161–9. doi: 10.1007/s00213-019-05445-3, PMID: 31927605

[29] Sabanovic M Lazari A Blanco-Pozo M Tisca C Tachrount M Martins-Bach AB . Lasting dynamic effects of the psychedelic 2,5-dimethoxy-4-iodoamphetamine ((+/-)-DOI) on cognitive flexibility. Mol Psychiatry. (2024) 6:1810–23. doi: 10.1038/s41380-024-02439-2, PMID: 38321122 PMC11371652

[30] Waltz JA . The neural underpinnings of cognitive flexibility and their disruption in psychotic illness. Neuroscience. (2017) 345:203–17. doi: 10.1016/j.neuroscience.2016.06.005, PMID: 27282085 PMC5143214

[31] Uddin LQ . Cognitive and behavioural flexibility: neural mechanisms and clinical considerations. Nat Rev Neurosci. (2021) 22:167–79. doi: 10.1038/s41583-021-00428-w, PMID: 33536614 PMC7856857

[32] Davis AK Barrett FS Griffiths RR . Psychological flexibility mediates the relations between acute psychedelic effects and subjective decreases in depression and anxiety. J Contextual Behav Sci. (2020) 15:39–45. doi: 10.1016/j.jcbs.2019.11.004, PMID: 32864325 PMC7451132

[33] Aust S Gärtner M Basso L Otte C Wingenfeld K Chae WR . Anxiety during ketamine infusions is associated with negative treatment responses in major depressive disorder. Eur Neuropsychopharmacol. (2019) 29:529–38. doi: 10.1016/j.euroneuro.2019.02.005, PMID: 30772118

[34] Timmermann C Zeifman RJ Erritzoe D Nutt DJ Carhart-Harris RL . Effects of DMT on mental health outcomes in healthy volunteers. Sci Rep. (2024) 14:3097. doi: 10.1038/s41598-024-53363-y, PMID: 38326357 PMC10850177

[35] Yaden DB Griffiths RR . The subjective effects of psychedelics are necessary for their enduring therapeutic effects. ACS Pharmacol Transl Sci. (2021) 4:568–72. doi: 10.1021/acsptsci.0c00194, PMID: 33861219 PMC8033615

[36] Yaden DB Earp BD Griffiths RR . Ethical issues regarding nonsubjective psychedelics as standard of care. Camb Q Healthc Ethics. (2022) 31:464–71. doi: 10.1017/S096318012200007X, PMID: 36398520 PMC9672929

[37] Olson DE . Biochemical mechanisms underlying psychedelic-induced neuroplasticity. Biochemistry. (2022) 61:127–36. doi: 10.1021/acs.biochem.1c00812, PMID: 35060714 PMC9004607

[38] Nielson EM May DG Forcehimes AA Bogenschutz MP . The psychedelic debriefing in alcohol dependence treatment: illustrating key change phenomena through qualitative content analysis of clinical sessions. Front Pharmacol. (2018) 9:132. doi: 10.3389/fphar.2018.00132, PMID: 29515449 PMC5826346

[39] Bogenschutz MP Podrebarac SK Duane JH Amegadzie SS Malone TC Owens LT . Clinical interpretations of patient experience in a trial of psilocybin-assisted psychotherapy for alcohol use disorder. Front Pharmacol. (2018) 9:100. doi: 10.3389/fphar.2018.00100, PMID: 29515439 PMC5826237

[40] Brizzi G Pupillo C Rastellic C Greco A Bernardelli L Di Natale AF . Cyberdelics: virtual reality hallucinations modulate cognitive-affective processes. Dialogues Clin Neurosci. (2025) 27(1):1–12. doi: 10.1080/19585969.2025.2499459, PMID: 40407250 PMC12107671

[41] Greco A Siegel M . A spatiotemporal style transfer algorithm for dynamic visual stimulus generation. Nat Comput Sci. (2025) 5:155–69. doi: 10.1038/s43588-024-00746-w, PMID: 39706876 PMC11860245

[42] Greco A Rastelli C Ubaldi A Riva G . Immersive exposure to simulated visual hallucinations modulates high-level human cognition. Conscious Cogn. (2025) 128:103808. doi: 10.1016/j.concog.2025.103808, PMID: 39862735

